# Introductory Lectures, Delivered at the University of Pennsylvania, November, 1839

**Published:** 1839-11-16

**Authors:** William Gibson

**Affiliations:** Professor of Surgery, &c. &c.


MEDICAL EXAMINER.

DEVOTED TO MEDICINE, SURGERY, AND THE COLLATERAL SCIENCES.

No. 46.] PHILADELPHIA, SATURDAY, NOVEMBER 16, 1839. [Vol. II.

INTRODUCTORY LECTURES, *delivered at the University of Pennsylvania, November*, 1839. By William Gibson, M. D., *Professor of Surgery*, &c. &c.

Lecture I.

Gentlemen,—It may be known to you, perhaps, that for several years I had contemplated a visit to Europe, for the sake of personal intercourse with the great surgeons and physicians of that country, and of comparing their establishments and practice with our own. Owing, however, to the difficulty of so disposing of professional business at home as to admit of the necessary period of absence, I found myself again and again disappointed, and obliged, reluctantly, to forego the pleasure and advantage I believed would result from the accomplishment of my views.

Fortunately, at the close of last session, I was enabled to make the long wished for arrangements, and, accordingly, embarked for Great Britain, where I had previously resided, and received the greater part of my professional education, but whose soil I had not touched for thirty years. You may easily imagine how eagerly I embraced, after a very short passage, the opportunity of flying to London, and throwing myself, as it were, into the arms of professional brethren, some of whom I had known in former days, and others only by their writings and reputation. Need I say, I was received *by all* with a kindness and disinterested liberality I had no reason to expect; for, having, with few exceptions, no claims upon their courtesy, save such as grew out of community of interest in the advancement of our science, it could not be supposed that the great professional men, engaged as they were in various pursuits in the metropolis of the world, would step out of their way for a solitary stranger, and give their time and attention, without certainty of receiving, in turn, an adequate compensation.

It will not be imagined for a moment, I trust, that I address you thus, from egotism, or vanity, or from ostentatious display of any attentions received, or advantages enjoyed, whilst abroad; so far from it, I feel bound, unreservedly, to declare my conviction that all such attentions were the result of desire, of those who bestowed them, to honour the country and our University, rather than the individual who happened, at the moment, to represent them. I will go further and say, I am persuaded that any respectable individual, from any of our well known medical establishments, who visits Europe, and calls upon distinguished professors and practitioners, will be received, almost invariably, in the kindest manner, and every demonstration afforded of desire to cultivate friendly intercourse, and to promote interchange of sentiment, opinion, and good offices. In fact, from all I have seen, I am convinced of the ardent wish, of the British especially, to acquire accurate information respecting our country, its institutions, civil and literary, its resources, population, and extent, its vast rivers, lakes, and mountains, its natural history, generally, and the physical and moral condition of its inhabitants,—most of whom they look upon as their own descendants, possessing the same spirit, energy, and habits, speaking the same language, and allied to them, closely, by the ties of consanguinity, and, as such, disposed to favour and cherish them, beyond all other foreigners, notwithstanding attempts made by some of their own travellers and writers, for interested purposes, to destroy their confidence, and alienate their affections.

After these preliminary remarks and acknowledgments, I may, with propriety, I hope, venture to furnish you a *sketch* of prominent medical characters, such as have come under my notice, during a sojourn of several months abroad, whom I have met in various capitals, in smaller towns and villages, or assembled, in large bodies, or associations, held, annually, at particular places. I wish, however, such account to be considered a *sketch merely*, or outline, to be filled up, or embodied, in the general course of lectures; for, having preserved, in form of journal, or diary, a full account of all I deemed worthy of notice, my manuscripts have amounted to several volumes, and contain a variety of details I could not have obtained from other sources than personal observation. To keep such a journal, I found it often necessary to rise at five, or six, in the morning, and seldom retired before twelve at night, and may safely say, perhaps, that little or nothing escaped me, and that I was enabled to form as good an estimate of characters and institutions, as if I had remained much longer abroad and pursued the ordinary course. Perhaps I may be permitted, also, to say, that from long experience, I could understand, at a *glance*, many professional matters another, differently situated, must have examined closely, to comprehend.

It was natural I should wish to see the Wellington of British surgery, as Sir Astley Cooper has been, emphatically, styled. I had attended his lectures, occasionally, and witnessed his operations, in Guy's and St. Thomas' hospitals, thirty years before; I was familiar with his writings and high reputation at home, abroad, and, indeed, throughout the civilized world, and felt no ordinary desire to form the acquaintance of one who, in addition to the highest professional renown, was allowed, by common consent, to be among the most finished gentlemen of the day;

I repaired, therefore, to his house, without any introduction whatever, was ushered into his presence, and received with a courtesy and urbanity I was totally unprepared to expect; for, upon my name being announced, he came forward with the ease and alacrity of a young man, and expressed, in the kindest possible way, his pleasure at meeting one connected with a university he had long known by reputation, and with some of whose professors he had been upon the most intimate terms of friendship, whilst fellow pupils with them, under the celebrated Hunter. Imagine to yourselves a tall, elegantly formed man, moderately robust, with a remarkably pleasing and striking countenance, red, and fresh as a rose, apparently about fifty-eight or sixty years of age, but, in reality, beyond seventy, very agile and graceful in all his movements, simply, but handsomely attired, with the ease and vivacity, and cheerfulness of a youth, with few or no marks of age, except a head as white as the driven snow, and you will be able to form a very just conception of the appearance of Sir Astley Cooper.

I had scarcely been seated five minutes before I found myself deeply engaged in discussing all the knotty points of surgery, question following question, in rapid succession, and the greatest interest evinced in the various answers returned—all touching points of practice, either peculiar to America, or in conformity with English or French doctrines, or notions, or, as sometimes happened, adverse to them. Thus employed, an hour glided quickly away, when a servant entered and whispered, audibly, that the rooms were full of patients, all anxious to obtain his advice. He rose suddenly, apologized for leaving me, and said, "come and breakfast with me to-morrow precisely at nine, and any morning, if you please, at the same hour, as long as you remain in London, and I will go through with you, day after day, the various preparations in my museum, the most valuable and choice of which are contained in my house." The next morning I was at my post by the appointed time, breakfast was served precisely to the minute, and half an hour afterwards I found myself in his museum listening to a lecture on the structure and functions of the thymus gland, illustrated by some of the most beautiful preparations I ever beheld. At half past ten I took leave, and Sir Astley said at parting, "come to me if you can, to-morrow at two o'clock, and I will take you to Guy's Hospital, show you the establishment and its large and splendid collection of preparations—many of which occurred in my own practice, and are very interesting and unique in their character." Whilst riding, upon that occasion, for miles along the crowded streets of London, and moving so slowly as scarcely to reach our destination for an hour and a half, I was forcibly struck with the fund of anecdote which he was constantly pouring forth, chiefly illustrative of the scenes of his long and eventful life, and relating, in many instances, to ludicrous, or remarkable, circumstances in the history of some of his professional brethren—all told in such a way, as to convince me that he possessed an innate love for fun, or mischief, so refined, however, by benevolence, as never to wound, or tarnish, the characters of those whose peculiarities, or infirmities, he pourtrayed. I was the more persuaded of this ingredient in his composition, afterwards, from hearing, through an old friend of his in the neighbourhood of Yarmouth, where he was born, the following anecdote—upon the truth of which I thought I could rely. "Sir Astley," said he, "was the son of a clergyman at Yarmouth, where, upon one occasion, the church bells began to ring, so loudly and vehemently, as to alarm the inhabitants, who ran in great numbers to the parsonage to inquire of the minister the cause of such terrific peals from the steeple. 'Oh!' said the reverend gentleman, 'I have no doubt it's all the work of that mischievous wag of mine—master Astley—and his hopeful playmate, Tom Goodfellow.' Accordingly, upon ascending the steeple, it was found, as predicted, that the boys were busily at work, full swing, pulling and hauling the rope in fine style, and amazingly delighted, at the stir and sensation they were creating throughout the town, and the trouble they were giving to the honest citizens."

During the ride Sir Astley mentioned to me, also, a striking peculiarity—which showed the power and extent of his memory—by remarking he could take up any of the poets, and from two or three readings repeat for years afterwards, whole passages without the slightest omission or mistake, and in proof of it, immediately recited several pages from Young's Night Thoughts. In conversing with him concerning the destruction of Hunter's papers, by Sir Everard Home, he remarked it was true, and an act of great folly on Sir Everard's part, inasmuch as it led to the belief he had never produced an original work, but had stolen every thing from Hunter, whereas, he had strong reason to believe, Sir Everard had only burnt papers which he conceived to be of little or no importance, and that he was not, justly, chargeable, in a single instance, with plagiarism. He also spoke of Home, as having been an excellent surgeon, full of information, devoted to his profession, but rough in his manners and operations, and so decided in character, and independent in views, as to give, upon many occasions, great offence to his patients and professional brethren.

Upon reaching Guy's Hospital, I had soon proof of the activity of Sir Astley's frame, and the vigour of his constitution; for he walked with the quickness of a young man, and was so rapid in his movements, as to render it difficult to keep pace with him. I was particularly struck with his demeanour towards the house surgeons, the pupils, the patients, the superannuated nurses, and every living thing about the establishment, his manner being as kind and conciliatory as possible, taking, in several instances, the old men and women aside, and inquiring into their wants, and, upon one occasion, going considerably out of his way, and up a long flight of stairs, expressly to shake hands with an old woman, who had been one of his principal nurses more than forty years before, and the only surviving individual, he said, who had been connected with the hospital as long as himself.

After showing several interesting cases in the wards—one, an amputation at the shoulder-joint, performed by Mr. Key, and in a fair way of recovery, the stump being nearly healed, and beautifully formed—he led the way to the surgical cabinet, and pointed out, with his own hand, each interesting specimen, giving its history and peculiarity, and waiting, patiently, until I had secured his remarks in my note-book. There, and afterwards at St. Thomas', I had the opportunity of examining all the preparations referred to in his great work on Hernia, the specimens in which the Aorta, the Iliacs, the subclavian and carotid arteries had been tied by himself, and the causes of failure, or success, amply demonstrated. There, also, I saw a specimen in which the subclavian had been tied, successfully, by Mr. Key, in a case where the axillary artery had been torn, in an attempt to restore a long-standing dislocation of the shoulder, and the result of which proved that I myself had been justified in pursuing the same course, under similar circumstances, long before. From the Museum, (the extent and beauty of which can only be appreciated by those who have examined it, closely, as I had frequently, afterwards, opportunity of doing, and of comparing each specimen with the printed catalogue, in shape of a large volume, prepared by the intelligent Dr. Hodgkin,) Sir Astley was kind enough to take me with him to the College of Surgeons, where we listened to a most eloquent discourse on the comparative anatomy of the kidney, in various animals, by the celebrated Mr. Owen, afterward introduced me to all the prominent surgeons and physicians present, and concluded by ushering me into the great Hunterian Museum, giving me free and unlimited access to every department of it, and there leaving me to revel in the regions of anatomical, surgical, and scientific research, to my heart's content. From that period I became a constant visiter at Sir Astley's, and, through him, formed the acquaintance of Sir Benjamin Brodie, and most of the other distinguished surgeons of London.

There are many, even in London, who believe Sir Astley to have retired from the profession, into the walks of private life. This is a great mistake; for although he has ceased, for some years, to perform the duties of a lecturer, and to attend at Guy's Hospital, except as consulting surgeon, he is still engaged in business and the examination of numerous cases at his own house. It is true he purchased, some years ago, a large farm near London, and intended to retire from the profession. For a time he was delighted with his agricultural occupations, but, at last, found himself so pursued into his retreat by his old patients, or so watched, and called upon, whenever he ventured to show himself in town, that he was obliged, in spite of himself, to resume his former pursuits, and has ever since attended, regularly, to the profession. Another circumstance, also, is said to have contributed to drive him from the country, which I have reason to believe to be well founded, inasmuch as the story was related to me, upon authority impossible to doubt. As long, said my informant, as Sir Astley could find a case of disease in his horses, cows, sheep or pigs, he was delighted and attended them with all the interest and fidelity he would have shown to a human being, often trepanned the head of some favourite ram, or ewe, in search of the cause of its disease, but the moment he found his stock in perfect condition, he at once became unhappy and sighed for his town house and the wards of Guy's Hospital.

Next to Sir Astley the most prominent London surgeon, perhaps, is Sir Benjamin Brodie, with whose writings and reputation I had long been familiar, but with whom, personally, I had no acquaintance during my first visit to Europe. I intended to treat him without ceremony, by calling and making myself known, but Sir Astley had anticipated me, by previously speaking in my favour, and afterwards presenting me with a letter to him. His appearance was altogether different from what I had supposed; for, instead of being full, stout and ruddy, as most Englishmen are, I found him thin, pale, and, seemingly, delicate and dyspeptic; the result, however, as it struck me, of hard professional work, mental as well as corporeal, rather than of natural feebleness of constitution. His countenance was pensive, and verging towards a melancholy cast, but the moment he spoke it was lighted up by a smile, so peculiarly winning and attractive, so strikingly benignant and intelligent, as (added to uncommon softness and sweetness of voice, with manners so gentle, unpretending and free from assurance or arrogance,) to be calculated, I thought, to captivate, irresistibly, the most fastidious taste. He inquired, eagerly, after our eminent men, most of whom he appeared to know, perfectly, by reputation, said he had been the intimate friend of our late Professor Dorsey, had corresponded with him for years, and formed the highest opinion of his talents and attainments.

After sitting sometime, and conversing, freely, on all topics, he invited me to accompany him to St. George's Hospital, where he may be said to have received his practical education, and of which great Hunterian school he has long been one of the principal surgeons and lecturers. Upon approaching the Hospital, a large and splendid edifice, at Hyde Park corner, I was surprised to find it present so different an aspect from the old building with which I was so familiar in former days, and could not conceive how it had been metamorphosed, until informed by Sir Benjamin that the original Hospital had been entirely demolished, and this new and splendid fabric reared in its place.

In walking the rounds of this new establishment, containing upwards of four hundred beds, I saw many diseased joints, and could not avoid asking Sir Benjamin if he performed as many amputations, for the relief of such diseases, as formerly. To which he replied "oh no—not the twentieth part." How then do you manage? By rest, position, splints and diet, was the answer. I told him I was delighted to hear so candid an avowal, inasmuch as I had long been in the habit in my lectures of condemning the numerous operations recommended in his work, and of substituting the simple and efficient remedies he had just mentioned, for a knowledge of which the profession was, chiefly, indebted to my countryman, the late Dr. PHYSICK. "Then (said he) you have not seen the last edition of my book, I will send it to you, and with it, that there may be no mistake in future, a full explanatory letter." These I afterwards received, and, in their proper place, will lay before you. After going through the whole hospital, prescribing for numerous patients, performing, in the wards, several simple operations, and explaining to the pupils the nature and peculiarity of each case, in a very lucid and unaffected way, Sir Benjamin took me to the *Museum*, consisting of a small, but very choice, collection of pathological specimens, beautifully prepared, and put up in better style than any similar collection I had seen in Europe.

Sir Benjamin Brodie may still be considered a young man, being only fifty-six, and in a climate where people attain great longevity and preserve their good looks, even in extreme old age, is likely to remain, for years to come, at the head of his profession, surrounded by crowds of patients and looked up to as one of the brightest ornaments of British surgery.

What medical man from this, or any other country, would visit London without seeing Mr. WILLIAM LAWRENCE, well known to the whole world, for the extent and variety of his information, for his intimate acquaintance with, and facility of speaking, most of the languages of modern Europe, for his celebrity as an anatomist and surgeon, for his excellent treatise on Hernia, for his lectures on comparative anatomy and zoology of man, for his beautifully written anatomical and surgical articles in Rees' Cyclopedia, and for his excellent character in private life?

Few, I will venture to say, that have formed his acquaintance but will bear testimony to his merits. I had not inquired about his personal appearance, and was, therefore, particularly struck, upon entering his study, with his fine, manly figure; his open, expressive, intelligent countenance; his large, and well proportioned head; his lofty and expanded forehead; his clear and brilliant complexion; his mild, but sparkling, grey eye: and then when he spoke in a tone so quiet, modest and unassuming, with a manner so gentle and conciliating, and expressed himself so kindly and affectionately towards our country—its institutions and citizens—I could not but feel I stood in the presence of a superior being, could almost imagine I had known him all my life, and warmed towards him, insensibly, as if he had been an old, long tried, and intimate friend. And, yet, at that very moment, he was heavily pressed by the hand of affliction, having, as he told me, lately lost a promising son, to whom he was uncommonly attached. The next day I saw him again, having met by appointment, and followed him, amidst a crowd of admiring pupils, through the large and numerous wards of *St. Bartholomew's* Hospital, where, in former days, I had spent many weary, but instructive hours, in listening to the discourses, and witnessing the operations of the celebrated Abernethy and his colleagues. Since that period there have been many additions to the good old building, many new wards, and others fitted up, and accommodated to the taste and fashion of modern times.

Mr. Lawrence made but few clinical remarks, in passing through the wards, but questioned each patient closely respecting his symptoms, and prescribed very carefully, and evidently took a deep interest in the fate of every sufferer. There were several fractured thighs, all treated by the inclined plane; numerous syphilitic affections, for which mercury, chiefly in form of blue pill, was administed; and several long standing cases of cancerous mamma, one of eleven years duration, for which no operations had been attempted, but only the most gentle palliatives employed—Mr. Lawrence remarking, "he had long known there were many cases of this description, which, if let alone, would not prove fatal for a long time, but if extirpated, would return speedily, and subject the patients to immense suffering and distress."

After several hours spent in the wards, Mr. Lawrence took me to the Hospital Museum, where I saw a splendid bust of the distinguished Pott; another of his grandson, Mr. Henry Earle, lately deceased; a third, and the best I had seen in England, of John Hunter, by Chantrey; and a very superb one of Abernethy, lately presented to the hospital by his widow. There was, also, a fine bust of Lawrence himself, and a great variety of beautiful anatomical and morbid preparations, many of them put up by Mr. Pajet, the curator of the museum, represented as a young man of uncommon talent and promise. By him I was shown three or four remarkable specimens of Ovarian cysts, removed successfully through a small opening in the abdomen. At the hospital I was introduced, by Mr. Lawrence, to Mr. STANLEY, one of the surgeons of that institution, and its lecturer on anatomy, a gentleman of distinguished talent, well known to the public for his zeal and acquirements, and from whom I received many marks of disinterested kindness.

Mr. SAMUEL COOPER has long been known in this country, in Europe, and, indeed, throughout the world, as a literary, scientific and practical surgeon, of the first eminence. Very early in life he cultivated, successfully, foreign languages, and was enabled, through the knowledge of these, to lay up stores of information, from which most of his brethren were cut off; and having such advantages, joined to peculiar taste, for minute surgical investigation, began by publishing the opinions and practice of eminent men of every country, with comments and illustrations of his own, so peculiarly just and appropriate, with remarks and criticisms, so fair, open and liberal, as to gain the confidence and respect of the whole profession, and secure for himself a reputation for probity, industry, talent, discrimination, learning and practical skill, and for such endearing qualities of the heart and gentleman-like manners, as few men, in any country or in any age, have ever attained. His great work, the *Dictionary of Practical Surgery*, and his *First Lines*, the fruits of his unrivalled industry, well known to every nation on earth, where surgery is cultivated and esteemed, would alone be sufficient to establish for him an enviable fame. But he is, also, well known by his valuable labours as professor of surgery in the University of London; by his practical skill as one of the surgeons to the London Hospital, and by great experience, acquired in military hospitals, and on the field of battle, during some of the most eventful periods of the Peninsular war. It was my good fortune to form the acquaintance, and enjoy the society of this gentleman, and to glean from him many valuable facts and observations, I could have obtained from few other sources. He is now about sixty years of age, rather below the middle stature; stout, muscular and of fine constitution, very mild and prepossessing in manners, and his physiognomy so peculiarly agreeable and benignant as to attract the notice of the most careless observer. He is still a most laborious student, and unceasingly employed in enlarging and improving the works from which he has reaped so abundant a harvest of renown. At his house I had the pleasure to meet several of his friends, more or less distinguished for their surgical writings; among the rest, Mr. Copeland, a surgeon, who for a long time has enjoyed high reputation in London, is well known, every where, by his works on the Rectum and Spine, and whom I found to be a most agreeable, lively, well educated man, full of information on all subjects connected with the profession and its collateral branches, and possessed of a fund of anecdote seldom met with among members of our profession in England, who, for the most part, are personages of very solemn demeanor, and generally measure their words and actions by the strictest rules of sobriety.

There are few surgeons in London better known and appreciated than Mr. Guthrie, and whose reputation abroad, especially on the continent of Europe, is so firmly established, not only as a spirited writer, but as a man of sound, practical information and experience, full of decision, so energetic and prompt in all his measures, so active and untiring in his habits, (qualities probably derived, in a measure, from long army service under Lord Wellington,) as to look, though now on the list of "grey-haired sires," speak and act like a boy.

Through the kindness of near relatives in this country, I was enabled to form his acquaintance and secure his friendship—witness his practice and operations in the *Westminster Hospital*, and his skill and dexterity in the management of cataract, and other diseases of the eye, to which he has devoted great attention in private practice; and as surgeon of the *Charing Cross Ophthalmic Institution*, where, just before leaving London, I saw him operate, with much skill and delicacy, for several diseases of the eye, and prescribe for more than eighty patients afflicted with all the varieties of ophthalmia, and other similar affections. In addition to these hospital duties, he delivers, annually, courses of lectures on surgery—is one of the examiners at Surgeons' Hall—enjoys extensive private practice among the higher classes of society, especially officers of the army and their connexions; and is so actively engaged from morning till night, as to render it difficult to imagine how he can find time to write books and pamphlets and lectures—and sufficient to account, notwithstanding his quickness and talent, for the carelessness of style, and occasional inaccuracy of matter evinced in his publications. In outline, Mr. Guthrie's face resembles that of the late Dr. Physick—his countenance is animated and expressive, and full of good humour and benevolence. He is, indeed, universally considered, I believe, to possess the most amiable feelings, but when roused by opposition, or cross-examined in courts of justice, is said to be so keen, searching, sarcastic and witty in his observations and replies, as to silence, in a short time, the most talented members of the bar. His stature is about the medium height—his form muscular, inclining to *embonpoint*, well turned, if not decidedly handsome, and his whole air and bearing lofty; but his manners, at the same time, so free, easy, engaging and devoid of affectation, as to gain, irresistibly, the confidence of strangers, and secure, in a short time, their attachment. Upon the whole I found him an honest, jovial, good-humoured, unprejudiced fellow, with all the solidity of an Englishman, politeness of a Frenchman, and the activity, independence, spirit and enterprise of an American, and was never better pleased than when I found myself in his company, surrounded by a score of his army companions, and could hear them talk over the many hard fought days of a peninsular campaign. Through Mr. Guthrie I was introduced to Sir James McGrigor, the celebrated medical director of the British army, who distinguished himself by long and meritorious services in India, and on the continent of Europe—not only as a most enterprising and energetic surgeon, but as an accomplished writer; who, in addition to these high qualities, secured for himself the esteem and admiration of the whole army, by the warmth of his heart and the uniform kindness he displayed towards his brethren. I need hardly say I was delighted with the urbanity and humility of this fine old gentleman, and, with much regret, was unable to join, through his invitation, the medical officers of the army at their annual dinner on the thirtieth of May.

Mr. Bransby Cooper, the nephew of Sir

Astley, holds a most respectable rank in London, as a lecturer on anatomy, as surgeon to Guy's hospital, and, as a practitioner, largely engaged in business. He is, comparatively, a young man, but has already published a valuable work on anatomy, and a volume on the ligaments, which have added much to his reputation. From having had the opportunity of knowing him intimately, and enjoying much of his society, I am enabled to speak, confidently, of his open and generous disposition, his frank and manly deportment and independence of character, combined with intelligence and substantial professional acquirement, rarely met with in the same individual. Like many other English surgeons, he spent the early part of his life in the army, and acquired considerable experience in the continental campaigns and Canada, during the last American war.

There is one of the London surgeons whose name I have not yet mentioned, but with whose reputation, I have no doubt, you are all more or less familiar—Mr. Liston, a native, and for years a resident of Edinburgh, and a near relative of Sir Robert Liston, formerly ambassador to this country. For weeks I had resided in London, visited all the great hospitals, had become more or less intimate with most of the great surgeons and physicians, and yet felt no curiosity to see Liston, because I had been told, not by his fellow practitioners, but by apparently disinterested persons, that he was full of eccentricity, very rough and uncouth in his manners, and a perfect ursa major, upon whose humour there could be no dependence; that, at best, he was a mere operator or carver, without judgment or discretion, and his knowledge of the treatment of disease, except by the knife, extremely limited. I left London, therefore, without seeing him. Returning, however, some weeks afterwards, it suddenly occurred to me, whilst passing his door, that it was wrong to be governed, in any case, by such prejudice. Under the influence of this feeling I pulled the bell, and, at the next moment, stood before him. He had been deeply engaged in examining the structure of an interesting pathological specimen, with a very splendid and powerful microscope, but rose, as I entered with an ease and gracefulness I had been unprepared to expect. You may judge of my surprise, indeed, when I found a tall, robust, and elegantly formed man approaching me, in whose handsome and regular features and penetrating eye, there was displayed a degree of intelligence, benevolence, modesty and playfulness combined, I had seldom before met with, which, joined to a manner peculiarly winning, unassuming and courteous, served at once to assure me that all the idle and gossipping tales I had listened to were mere creations of the fancy. It seemed to me as if he could read my thoughts, and was pleasing himself with my agreeable disappointment; for immediately after making myself known, he said, "come, sit down, you are the very man I want to see, I know all about you and your countrymen, and I hope you will not find me as bad a fellow as I have been represented." He then called my attention to his microscope, and the endless tortuosity of vessels displayed in the morbid structure under observation, and next took me into an adjoining room, a sort of *sanctum*, where I saw all sorts of recent preparations, some in process of maceration, some undergoing the bleaching operation, and others dried and ready for the case. In another room, the walls of which were covered with shelves, he pulled out numerous drawers filled with instruments, and said, "Here is my *clipper*"—meaning his cutting pliers—"there is my *bull dog*," putting into my hand, at the same moment, the prettiest and most efficient artery forceps I ever beheld, and so, in succession, showed me his entire collection, and then said, "now, if you have time, jump into my carriage, at the door, and ride to the North London Hospital, and I will show you my wards and patients." Accordingly we entered the magnificent coach, drawn by a pair of spanking bays, such as I had not seen in the queen's stables, and in a short time I found myself cheek by jowl with my new friend up to his elbows in hospital work, surrounded by a flock of students, just from a lecture at the London University, in the opposite square. I saw numerous injured limbs and ulcers all elevated upon inclined planes; ah, said I, these are old acquaintances of mine—you've heard, I perceive, of Physick and his plans. "Yes," he replied, "I told you I knew all about you." Then turning quickly around to one of his dressers, who had covered a wound with charpie spread with cerate, he said, "my dear fellow, what possible benefit can you promise yourself from that greasy, slouchy, plaster. Pray, if you love me, take it away,"—intending to remind the pupil of his practice of using *watery* instead of *unctuous* applications. During the visit he performed several minor operations with an ease and dexterity I have seldom before witnessed, and in several cases of disease, not requiring the knife, displayed uncommon skill and judgment, and proved himself equally versed in diagnosis. In a private room we found a respectable lady, her husband and daughter, who not meeting him at home, followed to the hospital to obtain his opinion respecting a cancerous mamma, and expressed strong desire to have it removed. He examined the breast very closely, and also the glands of the axilla, and finding the latter enlarged, immediately said, "my dear madam, do not suffer any one to touch you with the knife; let it alone and you may yet live for many years." The lady and her friends implored him to remove it, but he remained inflexible, and said, "if I cut it out it will return in three months, and you will die; if I let it alone you may live for a long time." It was just such a case as many a surgeon in Europe, and in this country, would have attacked by the knife without ceremony, and it gave me a better opinion of Liston's judgment and abilities than I should have formed, under other circumstances. I turned to him and said, my views correspond exactly with your own, but I am surprised at *your* giving such advice, inasmuch as you have the credit of never losing an opportunity of using the knife. He turned to his friend, Dr. Anthony Todd Thompson, and said "do you hear that?" and repeated my remark.

Such misrepresentations are, no doubt, to be traced, in some instances, to the apparent eccentricities of Mr. Liston, for though of seemingly robust frame and great strength of constitution, he is so solicitous of preserving his health, and is so confident of the value of active exercise on horseback, as for a long time to have kept hunters and a pack of hounds, which, while he lived at Edinburgh, he exercised at day-break, and long before most of his brethren were out of bed. It is said he has now abandoned the sport, having fractured his pelvis, and nearly broken his neck at some inordinate leap, and since that period has followed the exercise of a boatman on the Thames, by rowing every morning several miles before breakfast. He has a passion for domestic animals—horses, dogs, and cats. His enormous black cat, *Tom*, is almost as well known in London as Liston himself, being not unfrequently mounted along side of his master in the splendid chariot, and a constant guest at his hospitable board, where I had the honour of forming his acquaintance, by finding his foot in my soup before aware of its proximity to my plate.

Mr. Liston is about forty-five years of age, and though only a recent resident of London, has already much business, and will, no doubt, ere long, rise to the top of the profession. He is professor of clinical surgery in the London University, (an institution now nearly equal to that of Edinburgh, in the number of its pupils,) and has a large and valuable pathological cabinet, I took great pleasure in examining. He is the author, moreover, of an excellent work on surgery, rendered familiar to all by the American edition, with valuable notes, by Dr. Norris, of this city.

It was not my good fortune, whilst in London, to meet with the distinguished surgeon, Mr. Benjamin Travers, whom I knew formerly, and whose acquaintance I should have gladly sought again, but for domestic affliction which cut him off, for the time, from society. With great pleasure, however, I heard, from all quarters, of his high reputation, usefulness, and prosperity. Nor did I see, owing to my numerous engagements, surgeons Tyrrel, Green, or Key, the former the friend and pupil, the latter the nephew of Sir Astley Cooper, and all men of ability and reputation. At Guy's Hospital I was introduced, by Sir Astley Cooper, to Mr. Morgan, the house surgeon, who has a fair reputation, and has lately increased it by a valuable work on diseases of the eye.

With Mr. Alexander Shaw, the brother-in-law of my friend Sir Charles Bell, I was upon intimate terms, and found him extremely intelligent, well versed in all the departments of anatomy and surgery, possessed of the most amiable feelings, and greatly respected by the profession. Though quite a young man, he has already distinguished himself by various publications, and recently by a volume on the nervous system, which must add considerably to his well earned fame. That he will soon attain high rank in London as a man of science and as a practical surgeon, there can be no doubt. Of Mr. Herbert Mayo, one of the surgeons of Middlesex hospital, well known for his valuable publications, I saw much, and received from him many kind attentions. To Mr. Calloway I was made known by Mr. Bransby Cooper. He stands high as a surgeon, and is rapidly rising to eminence. The same may be said of Mr. Solly, one of the surgeons of St. Thomas Hospital, the author of a beautiful work on the brain, whose kind offices, politely proffered, I had much regret in not being able to avail myself of.

Of the many London physicians whose society I enjoyed, it would afford me great pleasure to speak. I have neither time, however, so to do, nor would it comport with the present occasion. But there is one gentleman of whom I cannot avoid notice, inasmuch as his name is associated with recent transactions in England, of so exciting a political character, as to have attracted the attention of the whole world. I allude to Sir James Clark, the physician of the queen, and as such, *disagreeably* involved in the affair of the Lady Flora Hastings. Of this affair it is needless to speak, as various conflicting accounts have reached every one, further than to express the opinion derived from intimate acquaintance with Sir James—though I never conversed with him on the subject—that it would have been impossible for such a man, so highly gifted, so mild, amiable, gentleman-like, so well versed in all the rules of high life and good breeding, and with all, so full of discretion, self-respect, and foresight, to have committed any of the enormities attributed to him for political purposes, and by writers of the vilest stamp and most degraded associations. That he may have been deceived by appearances, failed in his diagnosis, and suffered his judgment to be misled, by the fear of responsibility, or erred from various other causes, is probable, but that he lent himself, and professional reputation, to the vile purpose of blasting the character and ruining the happiness of an unfortunate female, to secure for himself, through court intrigue, favour and rewards, advantages he could not otherwise have gained, is an assertion I am sure his most virulent professional or political enemy can never seriously believe; and that he could explain to the entire satisfaction of the world, if circumstances would permit, his whole agency in the affair, I have the strongest reason, from disinterested sources, to assert.

In person Sir James is rather tall and slender, his countenance open and cheerful and pleasing, but marked with deep thought and reflection, and his accent slightly Scottish and agreeable. With manners highly polished and refined, the result of much travel and education, he gains the good-will and confidence of all who approach him, and leaves an indelible impression upon their minds, of integrity, talent, learning, taste, and benevolence. He is the author of an excellent treatise on consumption, and of another on climate, is engaged in extensive business, and more consulted in diseases of the chest than any physician in England. He is still the physician and intimate friend of the queen, and, except by a political party, is as much respected as any medical man in the kingdom. My last day in London was spent with his family, and the impression produced by their kindness and hospitality can never for a moment be effaced.

I have to regret that the limits of an ordinary introductory will not allow me to proceed with an account of other British surgeons and physicians whom I met, either at their places of residence in Edinburgh, Dublin, Bristol, or Norwich, including Sir Charles Bell, Sir George Ballingall, Professors Thompson, Allison, Christison, Graham, Hamilton, Drs. Abercrombie and Combe, Sir Philip Crampton, Mr. Carmichael, Drs. Obeirne, McDonnell, Kennedy, Maunsell, Houston, Professors Graves and Lendrick, Mr. John Hamilton, Dr. Pritchard, Mr. Estlin, Mr. Crosse, Mr. Dalrymple, and many others, or at the *Provincial Medical Association*, held at Liverpool, where I saw and formed the acquaintance of Drs. Hastings of Gloucester, Forbes of Chichester, Balfour of Bath, Simmonds of Bristol, Cowan of Reading, Bryce and Banning of Liverpool, Holm of Manchester, Brown of Sunderland, Baron of Cheltenham, Gregory of London, Mr. Turner of Manchester, Mr. James of Exeter, Messrs. Tudor, Soden and Norman of Bath, or at the *British Association*, at Birmingham, where I listened to the eloquent harangues of Mr. Hodgson, Mr. Knowles, Mr. Middlerose, Dr. Blackiston, Professor Macartney of Dublin, Drs. Hodgkin, and Arnott, and Professor Graham of London, Dr. Foville of Paris, and a host of other distingués, whose acquaintance or friendship I enjoyed. I hope, however, before the close of the session, to furnish you with some details of these gentlemen, and the institutions with which they are connected, and propose, in the mean time, to deliver a succeeding discourse, which will embrace the great surgeons, medical schools, and hospitals of Paris, as they now exist.

---

### Lectüre II.

From London, after a sojourn of many weeks, my steps were turned towards the French Capital, towards Paris—the wonder of the world, in all that relates to gaiety and fashion, to the fine arts and to sciences, to medical and surgical literature and practice, to military renown, and to historical and political associations, and revolutions, from the earliest periods down to the present time. With two or three exceptions, merely, I took no introductory letters, depending, as in England, mainly upon the voluntary courtesy and bonhommie of those whose acquaintance and society I sought—and was not disappointed. Through the kindness of an old Philadelphia friend,* remarkable for his excellent endowments and amiable character, long settled in Paris, and intimately acquainted with the *anatomy* of the place, its long, narrow, irregular streets, its splendid public buildings, its various shops and residences of celebrated men, the habits of the people, their singularities, and prejudices, and the modes of overcoming them, but, above all, for the interest he takes, as an amateur, in all that relates to the medical profession, especially where his American friends are concerned, I had abundant opportunities, by his guidance and assistance, of becoming acquainted, almost immediately, with the city and its concerns, which, without such valuable aid, I might have remained in for weeks and known very little about. With an ardour and enthusiasm I did not expect and had no right to calculate upon, he went with me from hospital to hospital, from surgeon to physician, from the Jardin Des Plantes to Pere la Chaise, from monuments to catacombs, from the Louvre to the site of the Bastile, and through alleys, holes and dungeons, where the light of heaven seemed not to have been admitted for half a century, hunting up books and prints and instruments, inquiring after anatomical and surgical preparations, and a thousand nondescript articles of value or curiosity which are only to be found in that extraordinary Capital. To several very intelligent young physicians, graduates of our school, now pursuing their studies, very diligently and successfully, at Paris, I felt myself, also, under great obligations for their kind attentions in going about with me to the different lectures, delivered often in very remote, and almost inaccessible places, and procuring information for me, on various subjects, I I should have found it very difficult otherwise to have obtained.*

Of the advantages of a residence in Paris for medical purposes, beyond most other cities, I was well apprized, but the real amount and value of such advantages I certainly had no adequate conception of until they were presented to my view, nor could I fully understand why pupils, after having completed their studies in this country, and sailed for Europe in quest of additional information, should, almost to a man, take up their quarters exclusively in the French metropolis, and never think afterwards of attending the lectures and walking the rounds of the English, Scotch and Irish colleges and hospitals. But I had not been a week in Paris before I understood, perfectly, the nature of the case, by finding that there were hundreds, nay thousands of individuals, employed in demonstrating, teaching and unravelling, in every possible way, the most intricate subjects, in every branch of our science and art, and for a compensation so exceedingly small, and oftentimes without any compensation at all, as to be within the limits of the poorest and most destitute student—that the demonstrations and lectures were carried on throughout every season of the year, and with an energy and enthusiasm, altogether surprising and unheard of in most other European countries—that *subjects*, owing to pe-

* Samuel I. Fisher, Esq.

* Drs. Bullock, Spencer, Grant, &c.

culiar regulations of government, an overgrown population and accidents, and diseases resulting therefrom, were more abundant and cheaper than elsewhere, and that *living*, where the student was really desirous of economising, and of employing his time to the utmost advantage, was so cheap as not to amount, necessarily, beyond a few francs a day. Above all, that the regular lectures in the different hospitals and institutions by men of the first eminence, were paid for by government, and *gratuitous*, as respected the pupil; that the reputation of these men was dependent, mainly, upon the exertions they made, in their several departments or lectureships, and that in turn, their chance of obtaining high standing in practice, and lucrative employment, was in proportion to their success and celebrity as professors, teachers, and hospital physicians, and surgeons; the natural consequence of all which was, that pupils would remain in Paris, where they had little or nothing to pay, and where the advantages were, at least, equal to those for which, in other countries, they would be obliged to pay, and that their teachers, from having the strongest possible motives for improving themselves and their classes, must, necessarily, acquire a skill and reputation, at least equal to that of teachers in other parts of the world.

My first visit upon reaching Paris, was to that quarter of the town called the Pays Latin, in which the greater number of the hospitals, the Ecole de Médicine and its museum, the clinical hospital of the school of medicine, the museum of Dupuytren, are situated, where all the medical students and many of the professors, private lecturers, demonstrators, medical booksellers, instrument makers, medical artistes, anatomical workers in wax and papier maché, preparers of natural and artificial skeletons and other varieties of surgical and anatomical specimens, reside; where the streets are so narrow and filthy, and without pavements or side walks, as to endanger life at every corner; where the houses are so high, old fashioned and gloomy as to resemble jails, or penitentiaries, and nearly shut out the light of heaven; where the Catacombs, those vast depositories of human bones, the accumulated collection of ages, lie beneath the feet, extend to unknown distances, and seem to respond, by hollow groans, to the tread of the foot passenger, and rumble beneath the jar of cumbrous vehicles and the tramp of clumsy animals, that are incessantly treading the narrow defiles, above their desolate, but populous, domains; where noisome smells of concentrated vigour and activity and varied odour, assail the olfactories from every quarter; where loud and discordant cries of wandering tribes of vagabonds vending their peculiar animal and vegetable productions, fall upon the sensitive and startled tympanum of the stranger like strokes of the sledge-hammer, or harsh gratings of the saw-pit; where the barking of dogs, the screams of parrots and the chattering of monkies, mixed with the gabble of old women and men; where the bowing and nodding, and scraping and salutations and recognitions of street passengers, bobbing against and shouldering each other—followed by the incessant and everlasting apology "Pardonnez moi Monsieur," and, in return, by the complacent shrug, and grin of the sufferer, and the exclamation "pas du tout," afford the most amusing and melancholy mixture of pleasurable and disagreeable sensation that can possibly be conceived, and have afforded, no doubt, many a scene for the dramatist and painter.

Yet in this very quarter, so different from the fine squares and buildings, and gardens, and broad avenues of other parts of Paris, and separated from them by the intervention of the river, are to be found the dwellings of men whose fame and reputation have extended to every corner of the earth; where the science of medicine in all its branches is taught with an assiduity and accuracy, enthusiasm and fidelity, unknown in most other parts of the world; where the student revels from dawn to sunset, and, if he please, throughout the night, among lectures, dissections, demonstrations and preparations, until he is stuffed and crammed, and saturated with knowledge to such extent, as to leave no room for additional supply; where he may go at almost any moment and witness important operations on the living body, listen to a lecture on the case and the reasons for performing it, and, if with an unfavourable result, have an opportunity of seeing the injection and dissection of the parts, and their mode of preservation; where he may perform with his own hand, operation after operation, guided by some able assistant, until he acquires a perfect knowledge of the principles which govern him, the instruments he employs, and the nature of the case in which he resorts to such measures; where, in short, he may be engaged for months, or years, in such varied and useful professional avocations as to be insensible to the disagreeable scenes by which he is surrounded, and to become so attached to the filth and inquinated atmosphere he has been digesting and respiring for so long a time, as to feel almost broken hearted at the prospect of leaving them.

It was in the midst of this professional region I found it necessary to establish my quarters; for, although I had attempted, whilst living near the palace, in the most fashionable part of the town, to follow the hospitals, by rising at day-break, I soon discovered it impossible to continue such long walks, without great fatigue and loss of time, and, therefore, fixed myself in lodgings, long celebrated as the resort of American students, and where I had the pleasure of being the inmate of some of my former pupils. Early one morning, whilst sitting in converse with these and my excellent friend professor Eve, of Georgia, there was a gentle tap at the door, followed by the entrance of one, at whose approach my friends simultaneously rose and bowed, in a way to indicate peculiar respect, and in the next moment, I found myself almost encircled by the arms of VELPEAU, who said, in the most complimentary way, he had called to pay his respects to me, and, immediately after, fixed his eye upon the tall, lathy, figure of one of my young countrymen, six feet three inches high, and remarked, in broken English and French, he perceived I had a *Kentuckian* in the room, much to the confusion, but amusement of my friend and his fellow students. I had often heard of Velpeau as a homely, ungainly personage, with grizzly hair standing up like a shoe brush, rough in his manners and careless in dress. I found him, however, polite, agreeable, lively, easy and genteel, dressed plainly, but with as much neatness as most other gentlemen. He sat for half an hour, conversing with great intelligence and good humour, on various subjects; asked numerous questions, respecting our medical men, and his former American pupils, whom he named and spoke of with pleasure. In referring to his numerous works, and expressing my surprise he should find time, engaged as he was, in hospital and private practice, to read and quote so many English, American and other foreign books, he replied with an honesty and candour I did not expect, "Oh, my dear Sir, you see how little I know of your language, it would be impossible for me to read all these books myself, but I have excellent young friends among your countrymen, and students from all parts of the world, and get *them* to read for me and furnish translations and extracts, and in this way, appear as learned as you have been pleased to consider me." I was delighted with this amiable frankness, and, afterwards, took every opportunity of seeing him at his house and at La Charité, where he is principal surgeon. His history is extraordinary, and calculated to make a strong impression upon a student who has experienced the hard usage and buffetings of this world, as it will convince him there is no situation in life, however humble, no circumstances however difficult, no misfortunes and entanglements, however complicated, he may not extricate himself from, and rise to highest eminence, provided he is endowed with talent, energy, enterprise and good conduct. I was walking with my old Philadelphia friend in the Palais Royal, in quest of a watch, and, struck with the open and honest physiognomy of a middle aged man, whom we observed, through the window, so busily engaged at his work as not to perceive us, determined to enter and examine his commodities. After selecting an article of beautiful workmanship, such as we had not seen in any other establishment, demanding the price, and then according to usage, endeavoring to get at the lowest sum, the man, with a deep sigh, and most disconsolate look, said that *his* profession was a most unfortunate one; that, for years, he had toiled from morning till night, poring over the wheels and springs of watches with magnifying glasses, until he had nearly put out a pair of the finest and sharpest eyes God ever made, and by long sitting, had injured his limbs and impaired his constitution. "Oh," said he, "that I had been a surgeon, how different might have been my situation!" Then turning, and looking us full in the face, he continued, "Gentlemen, I am a poor individual, without fame or consequence, but my history, inasmuch as it is connected with that of a dear friend, whose reputation is well known all over the world, is nevertheless a singular and interesting one, and, for his sake, if you can spare time, I will relate it to you." Struck with the manner and earnestness of the man, and favourably impressed towards him, we took seats in his small shop and listened to his narrative. "I was the son, said he, of a poor miller, and the father of my friend followed the occupation of blacksmith in the village of Breches and province of Loire, and, at an early age, we were both initiated in the mysteries of our paternal vocations, he shoeing horses and I grinding grain from morning till night. In spite, however, of the severe labour to which my friend was exposed, he devoted many hours of the night to improving his mind, and twice a week attended a country school three miles off. His father's library consisted of two books—the complete drover and a volume of medical receipts—which the young blacksmith was so enamoured of as to commit to memory, and, from that period, turned his attention to medicine. He continued, however, to shoe horses, and prescribe for their diseases, until his twenty-third year, when growing tired of such labour, and burning to distinguish himself in higher pursuits, proposed to me to leave our native village and repair to the Capital, where he was sure, he said, we should both meet with occupation worthy of our toil. With scanty means, and slender wardrobes fastened to our backs, we commenced our journey on foot, and after a time reached Tours, where the money of my friend giving out, he was obliged to remain and work at his trade, while I pursued my solitary way to the Capital, and meeting with no better employment took up with the villainous business of watch-making. Several weeks afterwards, my friend arrived, and hiring, for three francs, a black coat, which did not fit, and contrasted, strangely, with his country garments, waited upon the celebrated Dubois—offering to become his pupil—who, impressed, favorably, notwithstanding the ludicrous figure he cut in his long tailed coat and sky blue pantaloons, told him he might live among his servants and have the run of his kitchen, for some weeks, until he could ascertain the nature and extent of his qualifications. The proposal was joyously accepted, but before the expiration of the allotted time, my friend gave so many proofs of genius and talent, and worked with such assiduity and success as to astonish Dubois, and cause him, henceforward, to consider him as a companion and friend. From that moment the fortune of my village crony was made; for, under the excellent Dubois, he not only made astonishing progress in his medical studies, but was so diligent and untiring as to acquire, in a short time, such knowledge of the classics, and most of the languages of modern Europe, as to read them with facility. So much time, indeed, was devoted to all his pursuits as to render him very careless of his appearance and costume, and I remember how much mortification I experienced from perceiving that my master did not relish the occasional visits of my friend, and especially when he told me, upon one occasion, I ought to keep better company, for he was seriously afraid that ill-looking fellow would rob his shop. I endeavored after this, to prevail upon my old friend to attend better to his toilet, but he said such matters were beneath a man of science, and proofs of weak mind, and for his part, thought when a coat required brushing it was time to get a new one."

"Since that period only a few years have elapsed, and my country friend, farrier, and blacksmith, is now at the head of the profession in Paris, a distinguished professor and hospital surgeon, the author of large and valuable volumes in every department of the profession, and, withal, a man of fortune. And where, he continued, am I? Still a poor, miserable watch-maker in the Palais Royal, and the tenant of this pill-box of a shop, in which you are now sitting." And pray, Mr. Jarrosay, said I, may I ask who that friend of your's may be? "That friend, sir," said he, slowly rising from his bench, putting forth his right arm, and stamping firmly with his foot upon the floor, "that friend, sir, is no less than the celebrated Velpeau."

The next day I called upon Velpeau, and found him in his study behind a pile of books, which he was pitching, with great vivacity, from right to left, in search of authorities and quotations for a large work on surgery, then in press. He showed me the translation of a letter I had sent him, at his request, detailing the results of certain operations in my own practice, and said he had obtained similar documents from other American surgeons. Before leaving, I took the opportunity to ask if Jarrosay's story was correct. "Perfectly so, as far as it goes," said he, "he is still my friend, an honest man, and one of the best watch-makers in Paris, of whom you may purchase without hesitation." I returned to the Palais Royal, and secured the watch, and commend all in quest of such articles, "to go and do likewise."

I arrived in Paris shortly after the revolution of last May, when the hospitals were crowded with gun-shot wounds of every description, in men, women, and children, many of them received, accidentally, in their houses whilst engaged in domestic concerns, from stray bullets, which found their way into the most retired places. One poor fellow, among the rest, lost his life from a ball which struck him in the neck, whilst shaving in a garret of one of the lofty houses on the Quai Voltaire, and which had been fired from the opposite side of the river. Another, a noble looking man, while shutting the windows of his shop, received a shot in the middle of the thigh, which fractured the bone in a shocking manner. I saw him, among others, on the 11th of June, whilst accompanying Velpeau through his wards. He seemed in a deplorable condition, his skin like wax, and covered by clammy perspiration, his tongue foul, his eyes glassy, his system irritable in the extreme, his thigh prodigiously swollen, each orifice of the wound blocked up by a fungus, through the crevices of which offensive matter streamed copiously. Velpeau was evidently alarmed, and turning to me, said, "what do you think of that case, and what would you have done with it in your hospital?" I should have cut it off, I replied, as soon as possible after reaction. "Ah," said he "that would have given his limb no chance. I have been trying to save it; besides, he was unwilling to part with it; saying, he would rather die than wear a wooden leg. But I believe I must now operate, though it is rather late." Soon after, he repaired to his lecture room, and poured forth, extemporaneously, one of the most learned and interesting discourses, on spontaneous gangrene, I ever listened to, quoting immense number of authorities with the utmost ease and accuracy, but, in the midst of his fluency, suddenly stammered, fluttered, and floundered, and, under great embarrassment, said, "gentlemen, this is the first time I ever forgot a name,"—which was confirmed by the pupils around, some of whom had attended him for years. After lecture, the poor patient with broken thigh was brought in and placed upon the table, and Velpeau, with the long, narrow, double-edged knife, resembling a cut-and-thrust sword, and used by most French surgeons, amputated the limb, by forming a double flap, and sawing off the bone two inches above its shattered extremity. The poor fellow gave one loud and agonizing scream, and fell back. Velpeau, however, proceeded very deliberately and humanely, and, if not with the despatch of a Roux or Liston, with accuracy and neatness sufficient to prove himself, not only a learned lecturer, but a very clever operator. The man, through strength of constitution, recovered; but nine patients out of ten in a crowded hospital, like La Charité, would have died, and their lungs, and other internal organs, been found filled with metastatic abscesses.

There are persons, no doubt, in all parts of the world, ready to exclaim, upon hearing that Velpeau was a blacksmith, "Oh, he must be a vulgar fellow, it is impossible he can know anatomy, or surgery, or classics; he may be a good farrier, &c." Accordingly, his enemies in Paris, jealous of his reputation, avail themselves largely of the circumstance, and deride his claims to distinction. In these days, when people are springing, like mushrooms, out of the earth, and shooting, in a few hours, as it were, into notice, without any aid but their genius, can there be any thing more contemptible, weak, and ridiculous, than to depreciate them, and upon the very grounds upon which they are entitled to distinction? For we must all admit that one who, without family, without friends, without education, without means, and with nothing but his own native intellectual vigour and superiority to depend upon, can emerge from the cloud by which he is surrounded, and soar through regions of ineffable brightness, a shinng mark, to guide and control his high-born, rich, and college-bred brethren, should be an object of respect, sympathy, and affection, rather than obloquy, shame, and detestation. The proper reply, perhaps, to all boasters, who value themselves upon family distinctions, not the result of intellect, or of personal exertion, is that of the able and honest Pennsylvanian, who, though born in humble life, and bred a shoemaker, was enabled, by his genius and industry, to rise superior to the frowns of fortune, and by his own unaided efforts, to elevate himself to the highest rank in the councils of his country, and who, when jeered, twitted, and taunted in open debate, upon his low origin and humble occupation, told his assailant, in language which nearly cut him in twain, that "if *he* had been bred a shoemaker he would have been one still."

I have mentioned more than once the name of Roux, the chief surgeon of the *Hôtel Dieu*, and successor of the celebrated Dupuytren, the author of an interesting work on the comparative excellence of English and French surgery, and of other valuable publications, of whom you have all, no doubt, heard more or less. To see him in all his glory you must go to the scene of his exploits—the oldest and largest hospital in Paris, founded in the seventh century, containing, formerly, eight hundred beds, but now only six hundred, owing to a part of the hospital having recently been pulled down for the purpose of improving the streets on the southern bank of the Seine, the deficiency of which, however, is temporarily supplied by the use of an hospital in the Rue du Faubourg St. Antoine. I rose at five on a fine summer morning, the seventeenth of June, and, after a pleasant walk along the banks of the Seine, and through some of the antiquated streets of the neighbourhood, paid my first visit to the great school in which Desault and Bichat may be said to have lived and died; for the greater part of their lives were past in the Hôtel Dieu, in the hall of which I saw tablets with the most respectful and appropriate inscriptions to their memory, together with their portraits, and that of Dupuytren. For an hour and a half I walked through the long corridors, and wards, and rooms, and chapels of this venerable pile of buildings, founded by Bishop Landri, and added to, successively, by Philip Augustus, St. Louis, Henry the fourth, Louis the thirteenth, fourteenth, fifteenth and sixteenth; examined minutely the various arrangements for the reception of all classes of medical and surgical patients, and at seven found myself in presence of Roux, who had just entered the hospital, followed by a crowd of pupils, of every nation, colour, and appearance. He saw I was a stranger, and with great politeness immediately came forward, addressed me in broken English, mistaking me, probably, for a John Bull, but upon finding I was an American, expressed himself highly pleased, and invited me to examine with him every interesting case in the ward. I accompanied him from bed to bed, heard his remarks upon each patient, and, to my surprise saw him dress, with his own hand, every wound, ulcer, and fractured limb, and apply every bandage with a neatness and despatch almost incredible. I asked him if such was his habit. "Certainly," he replied, "I wish to give my pupils all the benefit of my experience and practice, for how could they learn from those in the hospital who are only in the act of learning themselves, and if a thing is worth doing at all, it is worth doing well?"

The appearance of Roux is rather singular. He is a dapper little gentleman of great stir and activity, straight as an arrow, stands bolt upright, and has a peculiar obliquity and twinkling of the eye, which indicates sly humour and self-satisfaction. His complexion is rosy and healthful, his nose thick, turned, slightly, to one side, and its extremity snub, and, somewhat, bulbous. In person, he is remarkably neat and particular, and seems not a little vain of his gentleman-like figure and manners. He is very kind, seemingly, to his patients, and I observed a peculiar smile of satisfaction playing over their features as he approached them, and in a good-humoured, chirping, way, said something to each, by way of pleasantry and consolation. I was much amused, in particular, with the familiarity of a rosy-cheeked, chubby little fellow, about twelve years of age, who, as Roux came near, shook his fist at him, and displayed all sorts of antics; towards whom Roux, in turn, seemed so attracted as to commence by tickling the varlet under the ribs, as he lay in bed, half naked, and ended by covering him with kisses. The French, indeed, are more familiar with their hospital patients, generally, than any other Europeans I met with—treating them, in many instances, more like companions and friends than strangers and dependants, and the patients, on the other hand, appear more intelligent and respectable, than the same class in England, or in this country, and seem to have an attachment to the higher orders, unknown elsewhere, unless in Ireland, where I saw much of the same kind feeling between the gentry and their servants, and medical men and their patients. In particular, I remember being struck by the bearing of Dr. Graves, of Dublin, towards his patients in the Meath Hospital, and could not help asking if, like Roux, he ever tickled them—which had the effect of tickling him not a little.

Roux has long been considered the neatest, quickest, and best operator in France, and although now verging towards sixty, appears to have lost none of his energy and activity of body or mind. His private practice is very large, and the labour he goes through, in the Hôtel Dieu, from seven, until ten, in the morning, is immense. I saw, in his wards, several amputated stumps, beautifully formed and nearly closed, through adhesion—also compound fractures of the leg, admirably adjusted, and put up with long, narrow splints, and the bandage of Scultetus. Notwithstanding, Roux is considered, in Paris, very unsuccessful in hospital practice. I cannot help thinking, however, from all I saw of this old hospital—the Hôtel Dieu—that its immense size and crowd of patients, the sluggish streams of the Seine nearly surrounding it, and exhalations therefrom, rising and filling it, together with the lofty houses in all the thickly settled streets in its vicinity, must tend, considerably, to curtail the number of successful cases, and that his failures are, mainly, owing to these and other causes, rather than want of skill, caution, and judicious after-treatment—as has been alleged.

Next to the Hôtel Dieu, the largest and best ventilated hospital in Paris, containing six hundred beds, and surrounded by spacious squares and gardens, filled with noble and luxuriant trees, is the *Hôpital de la Pitié*, situated in the Rue Copeau, and of which *Lisfranc* and *Sanson* are the chief surgeons, and *Serres*, *Clement*, and *Piorry*, the principal physicians. Sanson, at the time of my visit, was indisposed, having undergone lithotrity. I did not, therefore, see him; but of Lisfranc I saw enough to convince me that many of the reports concerning him are without foundation. He is a big, burley, narrow-shouldered man, more than six feet high, negligent in dress, awkward in gait, uncouth in manners, and loud and boisterous in discourse. In his lectures, he is said to be so unsparing of his brethren, and, in hospital practice, so harsh towards his patients, as to be unpopular with both. I cannot say whether these charges are well founded, but am inclined to believe them exaggerated, inasmuch as I saw nothing, beyond the natural want of polish in the man, (increased, I thought, by affectation of wishing to appear worse than he really was,) from which I should have drawn such a conclusion. I was seated, with a bevy of young medical friends, beneath the boughs of a wide spreading elm, on a delightful summer morning—the 18th of June—and saw him, for the first time, as he entered the hospital gate, and sauntered, slowly, along the gravelled walk of the long and wide avenue, leading to the ward containing his female patients. His head, covered with a rusty black and red cap, which, in shape of a tea-cup, stuck, like a plaster, to the summit of his crown—his long-waisted, scanty, snuff-coloured coat, dangling about his heels, and tapering away to sharpness, like the tail of a kite—his curiously contrived pantaloons, loose and bagging about his hips, and, at each stride, fluttering to the wind—his long, shovel-shaped shoes, scattering pebbles, as he walked, from right to left—his arms, standing out from his body, like the handle of a pump, conjoined with his outsretched, flexible neck, which swung, to and fro, beneath the pressure of his lengthy and wedge-shaped visage, presented one of the most ludicrous spectacles I ever beheld. He cast an inquiring, sidelong glance, at our group, as he passed, which seemed to indicate, I thought, a belief that we were amusing ourselves at his expense, for he instantly bristled up, and, with averted head, hurried out of sight. We followed, and, the next moment, saw him stretching through the wards, his right hand grasping a speculum, and his left a brush, for wiping the ulcerations after his instrument was applied. There were fifteen or twenty females labouring under the peculiar complaints, for the treatment of which he is so celebrated. Before commencing with these, however, he called the roll, to ascertain that all his *internes*, or house pupils, were mustered at their posts, and refused to proceed until a delinquent, who was in bed taking his morning nap, was brought to the scene of action. He then began a clinical discourse, explaining the general nature of the diseases before him, waxing warmer and warmer, as he proceeded, and gradually raising his stentorian voice, until its tones seemed to shake the foundations of the old building and startle the very rafters above our heads—whilst he, peering and scanning, from right to left, the looks of his auditors, with great self-satisfaction seemed to inquire into the effect, which his sesquipedalian words, and thundering sentences, produced upon their minds, and, after a few more sweeping oratorical flourishes, made a regular set-too at his patients—applying his instruments rapidly, and without ceremony—giving each pupil a fair opportunity to see, and judge, for himself.

From all I saw of Lisfranc upon this and other occasions, I am disposed to entertain a better opinion of him than the one usually held in France. That he is, naturally, an unpolished man, there can be no doubt; but full of information, practical skill, and judgment, equally certain; so much so, that even his enemies and professional rivals, admit him to be more successful, in most of his operations, than any one in Paris. My conviction, therefore, is—that he *assumes* many of the eccentricities of manner and dress, and puts on, for the sake of effect, the roistering and overbearing behaviour, for which he is so remarkable, rather in imitation of a Radcliffe or Abernethy, than from any want of kind feeling in his composition. Indeed, some of his friends spoke of him to me, as an honest, good-hearted fellow at bottom—full of waggishness and affected singularity, but a man of unquestionable erudition, science, and practical skill.

Ricord, considering his youth and the high position he holds as a lecturer and hospital surgeon, may be looked upon as one of the most eminent men in France. He is attached to the *Hôpital du Midi*, in the rue des Capucins, faubourg St. Jâcques, where he delivers clinical lectures several times a week, to crowds of pupils, on the various forms of syphilitic disease—his hospital being devoted exclusively to such complaints, under the management of himself, *Manec* and *Cullerier*; the latter of whom has, also, considerable reputation. Than Ricord, however, I have seldom listened to a more eloquent and successful lecturer—being remarkable for the simplicity and clearness of his language, which flows in a copious, uninterrupted stream, with an enunciation so distinct and emphatic as to be understood, with perfect ease, by every pupil and stranger. On this account, as well as his profound knowledge of the subject, his lectures are crowded with Englishmen and Americans, to the latter of whom he is particularly attentive—being, in fact, himself an American, born of French parents, in Baltimore, where he received his education, and resided, as he told me, until his sixteenth year. This is sufficient to account for his speaking English as fluently as French.

Ricord is now about thirty-five, a tall, fine-looking man, very prepossessing and gentlemanlike, and extensively engaged in private practice. In style and manner, he resembles FARRADAY, the celebrated London chemist—and best lecturer I heard in Britain.

It would be impossible, within the compass of an introductory, to speak of all the meritorious surgeons of Paris. I have, therefore, sketched a few only of the most prominent, and by these the rest may be judged and measured. There are two individuals, however, I cannot pass over—inasmuch as they have elevated themselves to the highest rank in the profession, and conferred an honour upon their country, and a blessing upon the world which time can neither destroy nor remove. I allude to CIVIALE and to LEROY D'ETIOLLES.

The history of lithotrity is too well known to require, in this place, any comment. It will be sufficient, therefore, to speak merely of the men to whom we are indebted for almost all our information on the subject. My acquaintance with Civiale commenced on the fifteenth of last June, at the *Hôpital Necker*, in the Rue de Sevres, of which establishment he is the chief surgeon. My old Philadelphia friend, as usual, accompanied me upon this occasion, and from having long been an *amateur* of lithotrity, and a particular acquaintance of the great operator, introduced me, and explained my anxiety to know him and witness his exploits. As Mr. Civiale is not less remarkable for kindness and hospitality than for skill and success as a lithotritist, it will create no surprise when I say that we were received with the utmost cordiality, and invited to accompany him through the hospital, after which we were taken to the lecture-room, and there found two or three calculous patients waiting for him. He commenced by examining one, upon whom he had previously operated several times, to ascertain if any fragment could be found. After repeated injections of the bladder, however, (with which he never dispenses) and very careful search, with a straight cannula and litholabe, not the smallest particle could be discovered, and the patient was discharged—cured. A second patient then presented himself, and, like the first, without assistance, got upon the table, when Civiale, after injecting the bladder, very cautiously and slowly introduced a *percuteur*, of construction peculiar to himself, caught the stone, instantly, and as quickly crushed it, again and again, by opening and shutting the instrument repeatedly, and taking fresh hold—without giving the slightest pain, for as soon as the instruments were withdrawn the man jumped off the table with the utmost alacrity, and with a smile upon his countenance, walked to his room. After this, Civiale commenced a lecture on lithotrity, which continued an hour, during which he explained, in the most minute and circumstantial way, the whole process, and particularly inculcated the importance of *injecting the bladder, and using the instrument with the utmost care and gentleness*, saying that all the accidents inexperienced operators had met with, were to be traced to harshness and violence. After lecture, we again walked with him through the wards, and saw numerous cases of strictures, enlarged prostates and other similar affections, all under treatment. Upon reaching the hospital gate, he insisted upon my friend and myself getting into his barouche, and allowing him to escort us home. During the ride, he talked chiefly of the frequency of stone in all parts of the world, said it was much more common than imagined; that many persons died from it who were never suspected, during life, to have had any complaint of the kind; that he had no doubt there were stone cases enough in many of the large American cities to employ two or three surgeons; that a young surgeon of Paris had found in Vienna more than three hundred cases of the disease in less than a year, when few or none were supposed to exist in that city, and concluded by remarking that he himself had met with stone in *a new-born* infant, which, upon analysis, was found to consist of three layers, each of peculiar composition. At parting, we received an invitation to dine with him a few days afterwards, saying, "I will then show you my whole collection of calculi, such as I have removed by the knife or reduced to fragments, and all the instruments I have ever found it necessary to employ."

Although I was prepared, from report, to meet with great dexterity on the part of Civiale, I had no just conception of the extraordinary facility with which he manœuvered his instruments, until I witnessed the operations referred to. There could not, indeed, have been exhibited more perfect skill in any branch of operative mechanics, than displayed by him upon the occasion, and yet not such as an *inexperienced* person would have estimated, for there was not the slightest attempt at *effect*, by twisting or turning of instruments, or any aim at feats of dexterity, but, on the contrary, the most deliberate, delicate, graceful movements imaginable, as if the instrument were performing its own evolutions, for it seemed to be *gliding along* under its own weight and power, rather than from any effort of the operator. This, indeed, is the great peculiarity of Civiale, and the foundation of his success; for most operators I had previously seen, hurried as much as possible, without proper regard to the patient's suffering, whereas Civiale watched closely the countenance, and when he saw any evidence of pain kept his instruments still, waited for some time, or removed them, if necessary. The last observation, indeed, he made to me, upon taking leave of him in Paris, was, "Be as gentle as possible, and do not keep the patient long on the table, and you will seldom experience any difficulty or disaster."

At the appointed time, accompanied by my friend, I waited upon Civiale, but found myself so engaged, in conversing with him and his guests, assembled to meet us, in enjoying his elegant hospitality—for he lives like a prince, in one of the most splendid and costly houses in Paris—as to have no opportunity of examining his instruments and calculi. After dinner we all retired to the drawing-room, in the middle of which stood a magnificent billiard table—to which the French gentlemen speedily made their way, Civiale among the rest, all anxious to display their skill. I felt curious to know how the great lithotritist would acquit himself in this kind of occupation, and, therefore, watched his manœuvres, closely, but was disappointed; for he proved himself unequal to his opponent, but bore his defeat with very good grace, whilst some of his friends, equally unsuccessful, appeared to be on wires and under the highest excitement. Upon one occasion, when Civiale had been making strenuous efforts to pocket the balls, I whispered to him I was sure he could *get them out* of the bag much easier than he could put them in, at which, understanding, at once, the professional allusion, he laughed immoderately. The next day I spent a long time with him in examining his superb collection of calculi and instruments, some of which last he insisted upon presenting to me—saying he wished them to be known to my class. From this period I saw Civiale daily, and witnessed numerous operations on his private patients, all which were performed, in a style, and with a result, impossible to exceed.

Some of you may, after all this, wish to know what sort of a looking man Mr. Civiale is. I answer, in few words, that he is one of the most polished, gentleman-like, and agreeable men I ever met, as simple and unaffected as a child, always good humoured, and, when he speaks, has a gracious smile playing over his features irresistibly attractive. He is about five feet eight inches in height, stout and muscular, very active, and handsomely proportioned. His features are regular, and expressive of great energy and decision, his eyes very penetrating, and his hair black as jet. He was a poor boy, without any resource but his genius. He is now one of the richest professional men in Paris—having received immense fees for his operations—is as liberal as he is rich, and, in every way, deserving of the reputation and wealth he has acquired. He is only about forty years of age, and, if he lives twenty years longer, will have quarried half the stone in Europe. To see and know him, and witness his performances, is, *alone*, well worth a trip across the Atlantic.

With Leroy D'Etiolles, the celebrated lithotritist, my acquaintance was not less intimate, perhaps, than with Civiale. Through the kindness of my friend Dr. Bertin, one of the most eminent physicians of Paris, I became known to him, and not only received at his hands great hospitality and kindness, but, through his unwearied assiduity in calling for me—frequently before six in the morning—witnessed a number of his operations on private patients, and in hospital practice, all which were performed with the utmost dexterity, and a success that, almost invariably, crowns his efforts. The first operations I saw him perform were on the 21st of June, at the clinical hospital of the School of Medicine, immediately after listening to an excellent lecture from *Cloquet*, in the same institution. His instruments differ, in many respects, from Civiale's, but are all intended to answer the same ends. *Leroy D'Etiolles*, indeed, is the inventor of a greater number of ingenious instruments, for many surgical purposes, besides lithotrity, than any other man, perhaps, in France.

In person he is rather spare, below the middle height, well formed, muscular, remarkably quick in his movements, and able, apparently, to encounter great professional labour. His features are large, prominent, full of animation and gayety, and indicate great quickness of thought and talent. He is a fine scholar, and writer, speaks English fluently, as well as other languages, is engaged in extensive practice, and enjoys the highest reputation, not only in Paris, but throughout Europe, for his share in advancing lithotrity. He is, moreover, an accomplished gentleman, admired and respected by all that form his acquaintance, and would hold high rank in the profession, and in society, in any part of the world.

It will appear, I trust, from all I have stated, that my visit to the metropolis of France must have proved to me, in every respect, most satisfactory. I take more pleasure in making this acknowledgment, because I had laboured, I confess, for years, under most erroneous impressions respecting it and its great professional men—impressions derived, in some instances, from foreign writers, and, in others, from reports of prejudiced Americans after their return home. Ocular demonstration, often the best corrective of error, has convinced me we have heard, and still hear, many statements, totally destitute of foundation, and others *true*, to a certain extent, which, properly explained, would lose half their importance, or admit, largely, of palliation. I have trespassed, however, too long, already, upon your time and patience, to think of discussing, upon the present occasion, topics which may, possibly, require, hereafter, ample investigation.

